# LncRNA‐TCONS_00026907 is involved in the progression and prognosis of cervical cancer through inhibiting miR‐143‐5p

**DOI:** 10.1002/cam4.1084

**Published:** 2017-05-23

**Authors:** Xuejing Jin, Xiangjian Chen, Yan Hu, Furong Ying, Ruanmin Zou, Feng Lin, Zhengzheng Shi, Xuejie Zhu, Xiaojian Yan, Shi Li, Hua Zhu

**Affiliations:** ^1^ Department of Obstetrics and GynecologyWenzhou Hospital of Integrated Traditional Chinese and Western MedicineAffiliated Hospital of Zhejiang Chinese Medical UniversityWenzhouZhejiangChina; ^2^ Department of General SurgeryThe First Affiliated Hospital of Wenzhou Medical UniversityWenzhouZhejiangChina; ^3^ Department of medicine laboratory centerThe First Affiliated Hospital of Wenzhou Medical UniversityWenzhouZhejiangChina; ^4^ Department of Obstetrics and GynecologyThe First Affiliated Hospital of Wenzhou Medical UniversityWenzhouZhejiangChina; ^5^ Department of UrologyThe First Affiliated Hospital of Wenzhou Medical UniversityWenzhouZhejiangChina

**Keywords:** Cell migration, cervical cancer, ELK1, LncRNA, MicroRNA‐143‐5p

## Abstract

Our previous long noncoding RNA (lncRNA) microarray revealed that lncRNA‐TCONS_00026907 is aberrantly expressed between cervical cancer tissues and adjacent tissues. This study aims to explore the potential role of TCONS_00026907 in the development of cervical cancer. The expression levels of TCONS_00026907 in cervical cancer tissues and adjacent tissues from 83 patients of cervical cancer were detected by quantitative real‐time polymerase chain reaction and the survival rate was analyzed. In vitro, HeLa and SiHa cells were transfected with small hairpin RNA (shRNA)‐TCONS_00026907, then cell proliferation, cycle distribution, apoptosis, migration and invasion were measured. To confirm TCONS_00026907 regulates expression of ELK1 through inhibiting miR‐143‐5p, overexpression of miR‐143‐5p and silencing of ELK1 were, respectively, performed in HeLa and SiHa cells. Results showed that TCONS_00026907 level was significantly higher in cervical cancer tissues compared to noncancerous tissues and the survival rate was lower in the high expression group. Silencing of TCONS_00026907, overexpression of miR‐143‐5p and silencing of ELK1 inhibited cervical cell cycle, proliferation, migration, and invasion, but promoted apoptosis, respectively. Furthermore, silencing of TCONS_00026907 suppressed the growth of cervical tumors and altered the expression of ELK1, p‐ELK1, C‐fos, Cyclin D1 and Bcl‐2 in vivo. Our study identifies TCONS_00026907 as a potent proto‐oncogene and indicates that TCONS_00026907/miR143‐5p/ELK1 regulatory pathway plays an important role in cervical cancer.

## Introduction

Cervical cancer is one of the most common gynecologic cancers and accounts for a large proportion of cancer fatalities among women worldwide. It has been reported that there are more than 529,800 new cervical cancer patients annually, and approximately 275,100 deaths due to cervical cancer each year 1. Current technology can reduce the incidence rate of squamous carcinoma, but the incidence rate of adenocarcinoma has greatly increased in recent years 2, 3. Studies have shown that adenocarcinoma has a poor prognosis because of the high rates of lymphatic metastasis and distant metastases and the low survival rate 4, 5. The most common therapeutic methods for cervical cancer are surgery, radiotherapy, and chemotherapy. However, due to a lack of effective biomarker screening and restricted diagnosis technology, cervical cancer continues to seriously affect women's health over the coming decades. The molecular and functional mechanisms of cervical cancer have not yet been fully identified. Therefore, an increased number of studies on the molecular targets of cervical cancer, such as noncoding RNA, is urgently necessary.

Long noncoding RNAs (lncRNAs), a kind of noncoding RNA longer than 200 nucleotides, can regulate gene expression through transcription regulation, posttranscription regulation, chromatin modification, and genomic imprinting 6, 7. Numerous studies have indicated that lncRNAs are closely related to tumor genesis, especially in liver 8, lung 9, and breast cancers 10, 11, 12. Additional research has shown that lncRNA participates in the cell cycle, cell differentiation 13, and apoptosis 14, 15. Therefore, lncRNA serves as an important therapeutic target in treating diseases. However, studies of the function and mechanisms of lncRNA in cancer are just beginning. The regulatory abilities of lncRNA are yet to be studied. Our study found that lncRNA‐TCONS_00026907 levels were higher in cervical cancer tissues than in adjacent noncancerous tissues.

MicroRNAs (miRNAs), a kind of noncoding RNA with about 20 nucleotides, participate in post‐transcriptional regulation by targeting the 3′‐UTR region of target genes 16, 17. Studies have revealed that miRNAs play an important role in various diseases, including cancer 18, 19. Additional researches have shown that some miRNAs are involved in the disease progression of cervical cancer 20, 21. miR‐143 has been studied in gastric cancer 22. However, the function and mechanisms of miR‐143‐5p in cervical cancer are still unclear.

ELK1 is a member of the Ets family of transcription factors and of the ternary complex factor (TCF) subfamily. Studies have revealed that ELK1 is associated with prostate cancer 23 and takes part in c‐fos, MAPK signaling pathways, and the immune system 24. However, the role of ELK1 in cervical cancer has yet to be studied.

When compared with previous studies, our microarray assay results indicated that the level of TCONS_00026907 is significantly higher in cervical cancer tissues. We also found that TCONS_00026907 negatively regulates miR‐143‐5p, and miR‐143‐5p negatively regulates ELK1. Therefore, we speculated that lncRNA‐TCONS_00026907, which can serve as a competing endogenous RNA (ceRNA), is involved in the progression and prognosis of cervical cancer though the inhibition of miR‐143‐5p and the promotion of ELK1. Thus, lncRNA‐TCONS_00026907 can be a potential biomarker in the diagnosis of cervical cancer.

## Materials and Methods

### Clinical specimens

For this study, we collected tissue samples from 83 patients with cervical cancer in the First Affiliated Hospital of Wenzhou Medical University between 2011 and 2013. Each patient provided informed consent. All tissue samples were saved at −80°C.

### Cell lines

Human cervical cancer cell lines (HeLa and SiHa) were purchased from American Type Culture Collection (ATCC, USA). HeLa and SiHa cells were grown at 37 °C in an appropriate incubator containing 5% CO_2_ in a RPMI 1640 medium (Invitrogen, USA) including 10% fetal bovine serum (FBS; Gibco, USA), penicillin (100 U/mL), and streptomycin (100 *μ*g/mL).

### Lentiviral vector construction, production and transfection

Human TCONS_00026907 full‐length cDNA was amplified by PCR from the mRNA of HeLa cells. Then, the shTCONS_00026907 sequences were designed. The sequence for shRNA‐83 is 5′‐GCA CAT TCT GCC CTG ATT TCC‐3′; the sequence for shRNA‐1373 is 5′‐GCC TTT CCC TGC TAC TTG TGT‐3′; the sequence for shRNA‐1593 is 5′‐GCC TTT GGA AGC TCT TGA AGG‐3′; and the sequence for shRNA‐1691 is 5′‐GCA CAG AGC AAC TCT ATA ATA‐3′. The sequence for shLuc is 5′‐TGC GCT GCT GGT GCC AAC CCT ATT CT–3′. shLuc was used as the negative control (NC). The objective products were cloned into pcDNA3.1 (Invitrogen, USA). The constructed vectors and the lentivirus packaging vectors (pMD2.G, pMDL‐G/P‐RRE, pRSV‐REV) were cotransfected into HeLa and SiHa cells for 48 h, respectively. Lentiviruses were produced, harvested, and purified with ultracentrifugation. HeLa and SiHa cells (10,000 cells/well) were seeded in 24‐well plates and transfected with lentivirus, using 8 *μ*g/mL polybrene (Sigma, USA). Stable expression cells were screened in a medium containing 800 *μ*g/mL G418 (Sigma, USA).

### Quantitative real‐time reverse transcription PCR (qRT‐PCR)

According to the manufacturer's instructions, The total RNA was isolated using the TRIzol reagent (Invitrogen, USA). A quantity of 1 *μ*g of total RNA was reversely transcribed with random primers, using the RevertAid First Strand cDNA Synthesis kit (Thermo Fisher Scientific, USA). As described previously 25, the SYBR‐Green PCR Master Mix kit (Takara, Japan) was used to measure the mRNA expression levels of TCONS_00026907, miR‐143‐5p, and ELK1. The primer sequences for GAPDH (internal control) are 5′‐CCT CGT CTC ATA GAC AAG ATG GT‐3′ (forward primer) and 5′‐GGG TAG AGT CAT ACT GGA ACA TG‐3′ (reverse primer). The primer sequences for TCONS_00026907 are 5′‐TGG ATT GTT GGG TAT ATT TTG GA‐3′ (forward primer) and 5′‐TGT ATG AAG AGG ATG CTG AAG GC ‐3′ (reverse primer); The primer sequences for hsa‐miR‐143‐5p are 5′‐GGTGCAGTGCTGCATCT‐3′ (forward primer) and 5′‐CTC AAC TGG TGT CGT GGA‐3′ (reverse primer). The primer sequences for hsa‐ ELK1 are 5′‐TAG CGA ATC AAT CCG TGG CG‐3′ (forward primer) and 5′‐CCC GTG AAG TCC AGG AGA TGA‐3′ (reverse primer). The primer sequences for U6 are 5′‐CTC GCT TCG GCA GCA CA‐3′ (forward primer) and 5′‐AAC GCT TCA CGA ATT TGC GT ‐3′ (reverse primer). All data are displayed as the mean ± SD of three independent experiments.

### Protein extraction and Western blot

HeLa and SiHa cells, transfected with shTCONS_00026907 (shRNA and NC), and the treated BALB/c athymic nude mice tissue samples were lysed using a lysis buffer, including a protease inhibitor cocktail (Sigma‐Aldrich, USA). The proteins in equal concentrations were separated by 8% SDS/PAGE gels and transferred to PVDF membranes (Millipore, USA). The primary antibodies were used to incubate the PVDF membranes with the target proteins at 4°C overnight. The HRP‐conjugated secondary antibodies were used to incubate the treated PVDF membranes for 1 h at room temperature. The primary antibodies used in our study were the anti‐ELK‐1 (1:1000, Santa Cruz Biotechnology, USA)), the anti‐p‐ELK1 (1:1000, Santa Cruz Biotechnology, USA), the anti‐C‐Fos (1:1000, Santa Cruz Biotechnology, USA), the anti‐Cyclin D1 (1:1000, Beijing Genomics Institute, China), and the anti‐Bcl‐2 antibodies (1:200; Abcam, USA). The anti‐GAPDH antibody (1:4000, Beverly, USA) was used as an internal control.

### Hematoxylin and eosin stain (H&E) staining

Tissues were fixed in 10% formalin and processed for paraffin embedding. Paraffin embedded sections were cut at 5 *μ*m and stained with hematoxylin and eosin to assess the histological changes.

### Immunofluorescence (IF) staining

HeLa and SiHa cells (10,000 cells/mL), transfected with shTCONS_00026907 (shRNA and NC), were cultured on a coverslip. Briefly, the treated HeLa and SiHa cells were fixed with 3.7% paraformaldehyde in PBS for 20 min at room temperature. 0.2% Triton X‐100 solutions in PBS were used to permeabilize cells for 10 min at room temperature. Then, cells were incubated with the primary antibody of p‐ELK1 (1:100, Santa Cruz Biotechnology, USA) overnight at 4°C. After being washed, the fluorescence‐conjugated secondary antibody (goat‐anti‐rabbit‐Alexa 594‐conjugated antibodies, Life Technologies, USA) was used to incubate the treated coverslips for 1 h at room temperature. The treated coverslips were then incubated with 4′,6‐Diamidine‐2′‐phenylindole dihydrochloride (DAPI; Life Technologies, USA) for 10 min at room temperature. The images were obtained using a fluorescence microscope (Olympus, USA).

### Immunohistochemistry

Tissue sections were rehydrated and incubated with anti‐ELK1 antibody overnight at 4°C, followed by incubation with a horseradish peroxidase‐conjugated secondary antibody for 30 min at room temperature. Visualization was conducted, using diaminobenzidine as the HRP substrate.

### Luciferase reporter assays

For the luciferase reporter assay to confirm that miR‐143‐5p was targeting ELK1, HeLa cells were transfected with miR‐143‐5p mimics, negative control mimics, and miR‐143‐5p mimics plus luciferase reporter vectors expressing wild ELK1 3′UTR or mutant ELK1 3′UTR, or NC mimics plus luciferase reporter vectors expressing wild‐type ELK1 3′UTR or mutant ELK1 3′UTR using Lipofectamine^™^ 2000 (Invitrogen, USA) according to the manufacturer's instructions. To confirm TCONS_00026907′s influence on miR‐143‐5p, HeLa cells were transfected with miR‐143‐5p mimics plus luciferase reporter vectors expressing the wild TCONS_00026907 sequence or the mutant TCONS_00026907 sequence, or NC mimics plus luciferase reporter vectors expressing the wild‐type TCONS_00026907 sequence or the mutant TCONS_00026907 sequence. After the transfections for 48 h, the luciferase activities were measured, using a Dual‐Luciferase reporter assay system (Promega, USA) as the manufacturer's instructions.

### Proliferation assay

HeLa and SiHa cells (50,000 cells/well), transfected with shTCONS_00026907 (shRNA and NC), were seeded in a 96‐well plate and were incubated at 37°C. At 24, 48, and 72 h, respectively, 100 *μ*L of CCK8 solution (Dojindo Molecular Technologies, Japan) was added to each well. After 4 h, a micro‐plate reader (Bio Tek Instruments, USA) was used to measure the absorbance at 450 nm.

### Migration and invasion assays

Twenty four‐well Millipore transwell chambers (Millipore Corporation, USA) were used to perform the migration and invasion assays. For the migration assay, the treated HeLa and SiHa cells (1 × 10^5^ cells/well) in a serum‐free medium containing 0.1% bovine serum albumin were added to the top of each well, and 600 *μ*L of RPMI 1640 medium containing 10% FBS as a chemoattractant were added to the lower chamber. After 24 h, the migratory cells were fixed with 4% paraformaldehyde for 20 min, and stained with crystal violet solution (Sigma‐Aldrich, USA) for 20 min. The migrated cells were photographed and counted. The data were shown as the mean value of cells per field. For the invasion assay, the diluted matrigel (BD Biosciences, USA) paved the upper well of the transwell chamber and was incubated for 1 h at 37°C. The other steps are the same as the migration assay.

### Flow cytometric analysis of the cell cycle

The treated HeLa and SiHa cells were harvested, fixed in 70% ethanol, and stained with propidium iodide solution (50 mg/mL propidium iodide, 50 mg/mL RNase A, 0.1% Triton‐X, 0.1 mmol/L EDTA). The images of the cell cycle were obtained and analyzed using the FACS Calibur (BD Biosciences, USA) and the Flowjo software (Tree Star Corp, USA).

### Flow cytometric analysis of the cell apoptosis

Likewise, the treated HeLa and SiHa cells were harvested and stained with FITC‐Annexin V and Propidium iodide (PI). The images of cell apoptosis were obtained and analyzed, using the FACS Calibur (BD Biosciences, USA) and the Flowjo software (Tree Star Corp, USA).

### Tumor formation in nude mice

The nude mouse tumorigenicity assay was approved by the Institutional Committee and carried out based on the Institutional Animal Care and Use Committee's guidelines. The treated HeLa and SiHa cells (1 × 10^7^ cells in 100 *μ*L) were subcutaneously injected into 5‐week‐old BALB/c athymic nude mice. At specific times, the mice were killed and their tumor volumes were measured.

### Statistical analysis

All data are expressed as the mean ± SD. The statistical significance was set at *P* < 0.05. Comparison between groups was analyzed by a Student's *t* test or one‐way analysis of variance (ANOVA). The survival rates were analyzed by the Kaplan–Meier estimator. All data were made by IBM SPSS Statistics 21.

## Results

### LncRNA‐TCONS_00026907 overexpression in human cervical cancer tissues

**Figure 1:**
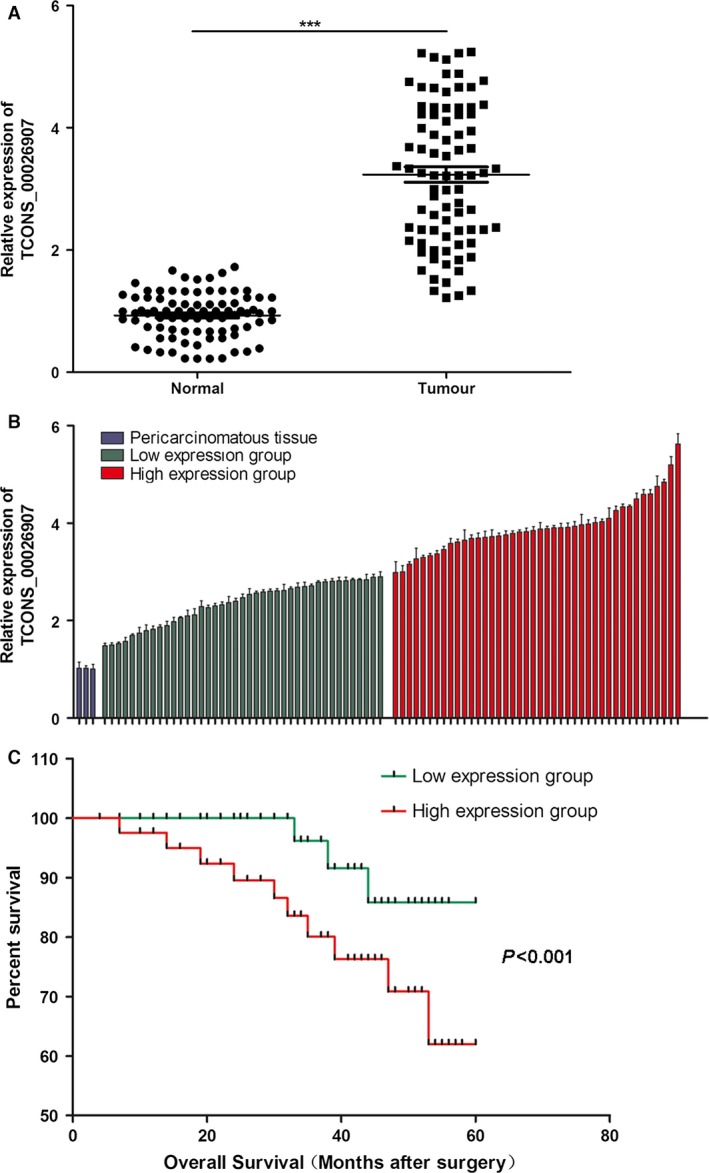
LncRNA‐TCONS_00026907 is overexpressed in cervical cancer tissues and related to the survival prognosis of patients with cervical cancer. The cervical cancer tissues and adjacent normal tissues from 83 patients with cervical cancer were collected. (A) Quantitative real‐time PCR results showed that TCONS_00026907 is overexpressed in cervical cancer tissues compared to adjacent normal tissues (*n* = 83, ***P < 0.001). (B) The expression of TCONS_00026907 was presented as fold‐change in tumor tissues relative to normal tissues. The blue column is defined as TCONS_00026907 expression in three normal tissue samples, while the green and red are defined as TCONS_00026907 expression in 83 tumor tissue samples. Patients were classified into two groups according to the median ratio of relative TCONS_00026907 expression in tumor tissues: a relatively low‐TCONS_00026907 group (*n* = 41, green column) and a relatively high‐TCONS_00026907 group (*n* = 42, red column). (C) The Kaplan–Meier survival analysis on the basis of TCONS_00026907 expression in 83 patients with cervical cancer. Patients in high‐TCONS_00026907 group has a poor survival prognosis.

To study the expression of lncRNA‐TCONS_00026907 in human cervical cancer tissues, we detected their mRNA expression levels in 83 cases of human cervical cancer tissues and adjacent noncancerous tissues. The results indicated that the TCONS_00026907 expression level was higher in cervical cancer tissues than in adjacent noncancerous tissues (*P* < 0.001) (Fig. 1A). According to the expression level of TCONS_00026907, we divided cervical cancer patients into two groups. The high expression group contained 42 patients and the low expression group contained 41 patients (Fig. 1B). Based on the mRNA expression levels of TCONS_00026907 in 83 patients with cervical cancer, we further analyzed the probability of survival for patients with low expression levels of TCONS_00026907 (*n* = 41) and high expression levels of TCONS_00026907 (*n* = 42). We found that the survival rate was lower in high expression group than in the low expression group (Fig. 1C). The high expression group displayed larger tumor size (*P* < 0.001), a higher average tumor node metastasis (TNM) stage (*P* < 0.001), and a stronger possibility of metastatic cancer (*P* < 0.001) when compared with the low expression group (Table 1).

**Table 1:** Correlation between TCONS_00026907 expression and clinicopathological characteristics in patients with cervical cancer

Clinical parameter	Number of cases (%)	TCONS_00026907		Chi‐squared test *P*‐value
		High expression group no. of cases	Low expression group, no. of cases	
		42	41	
Age (years)				0.1516
<30	3 (3.6)	1	2	
30–55	59 (71.1)	30	29	
>55	21 (25.3)	11	10	
Age at first birth(years)				0.8369
<18	4 (4.8)	3	1	
18–24	32 (38.6)	17	15	
>24	39 (47.0)	18	21	
nulliparous	8 (9.6)	4	4	
Cesaren section				0.1435
Never	70 (84.3)	33	37	
Ever	13 (15.7)	9	4	
Tumor types				0.6192
Endophytic type	16 (19.3)	8	8	
Ulcerative type	17 (20.5)	11	6	
Endocervical type	11 (13.3)	5	6	
Exophytic type	39 (46.9)	18	21	
Stages				0.0012
I a	9 (10.8)	1	8	
I b	20 (24.1)	6	14	
II a	54 (65.1)	35	19	
Size(cm)				0.0012
<4	32 (38.6)	9	23	
≥4	51 (61.4)	33	18	
Lymphatic metastasis				<0.0001
Negative	57 (68.7)	20	37	
Positive	26 (31.3)	22	4	
HPV16/18				0.5914
Negative	14 (16.9)	8	6	
Positive	69 (83.1)	34	35	

### Silencing of TCONS_00026907 expression by lentivirus inhibits the progression of cervical cells

**Figure 2:**
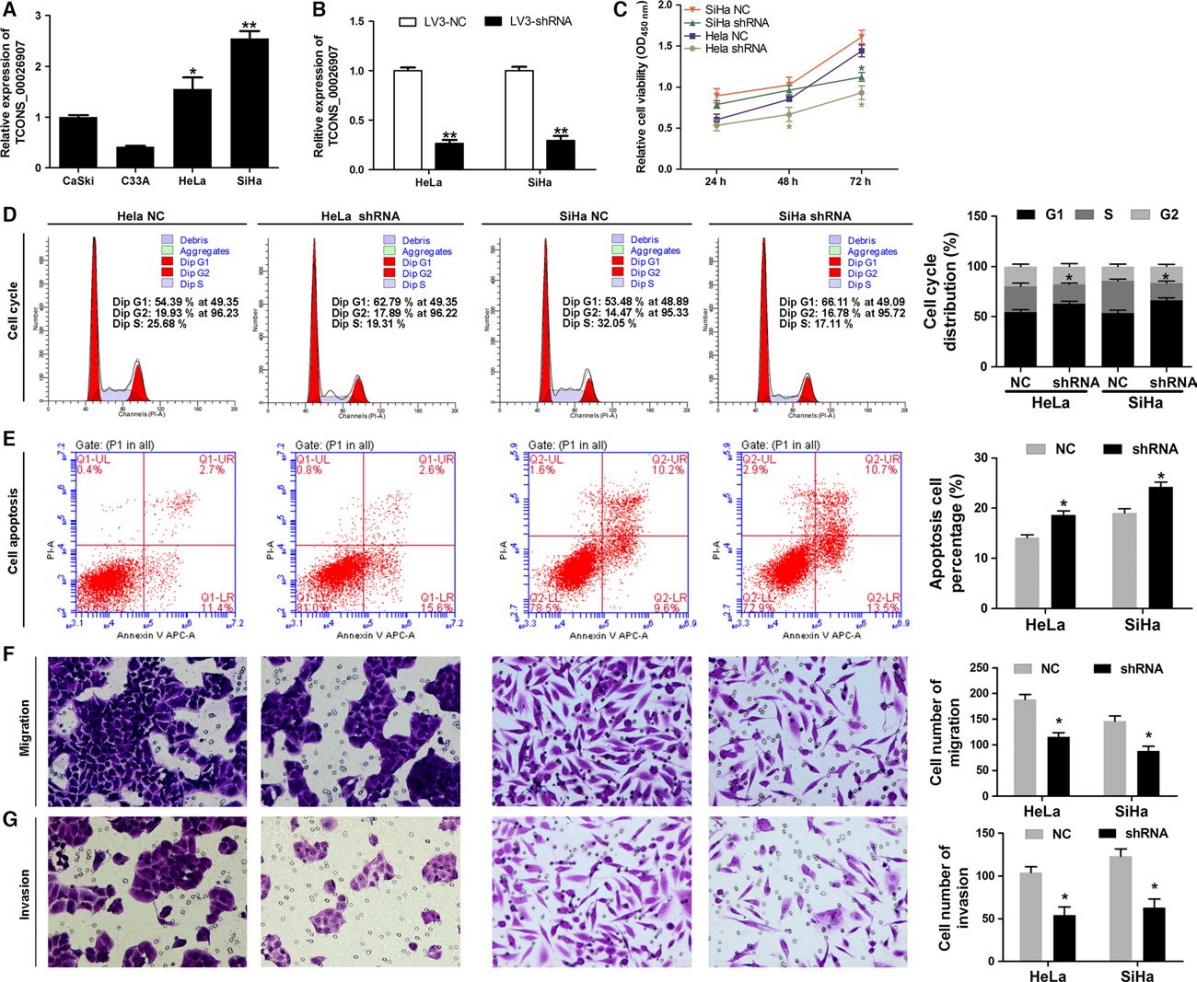
Silencing of TCONS_00026907 by lentivirus inhibits the progression of cervical cancer cells. (A) Quantitative real‐time PCR was used to detect the expression level of TCONS_00026907 in epidermoid cervical carcinoma cells (CaSki) and cervical cancer cell lines (C33A, HeLa, and SiHa). HeLa and SiHa cells were selected and transfected with negative control (NC) or shRNA (shRNA: targeting TCONS_00026907). (B) A reduction in the level of TCONS_00026907 in HeLa and SiHa cells transfected with shTCONS_00026907. The mRNA expression of TCONS_00026907 was measured by Quantitative real‐time PCR (**P < 0.01). (C) Result of CCK‐8 assay revealed that proliferation of HeLa and SiHa cells is inhibited by silencing of TCONS_00026907 (*P < 0.01). (D) Silencing of TCONS_00026907 induced cell cycle arrest at G1/S phase (*P < 0.01). The cell cycle distribution was detected by flow cytometry. (E) Apoptosis of HeLa and SiHa cells transfected with shTCONS_00026907 increased (*P < 0.01). Measured by Annexin V‐FITC/PI staining and flow cytometry. (F and G) Number of migrated and invaded cells was increased by shTCONS_00026907 (*P < 0.01). Migration and invasion were measured by Transwell assay, 200× magnification.

The expression level of TCONS_00026907 was detected by qRT‐PCR in epidermoid cervical carcinoma cells (CaSki) and cervical cancer cell lines (C33A, HeLa, and SiHa). Our results indicated that the TCONS_00026907 expression levels were higher in HeLa cells (*P* < 0.05) and SiHa cells (*P* < 0.01) than in CaSki cells (Fig. 2A). In order to study the regulatory mechanism of TCONS_00026907 in cervical cancer, lentiviral vectors of shTCONS_00026907s (lv3‐shRNA and lv3‐NC) were constructed and transfected into HeLa and SiHa cells. The qRT‐PCR results showed that the expression level of TCONS_00026907 was downregulated in HeLa and SiHa cells transfected with shRNAs of TCONS_00026907 when compared with the negative control (*P* < 0.01) (Fig. 2B). The proliferation ability of HeLa and SiHa cells decreased when they were transfected with shRNA when compared with the NC (Fig. 2C and D). TCONS_00026907 silence‐induced cell cycle arrest in HeLa was measured by flow cytometry. This indicates that the growth‐inhibiting effect of lenti‐shTCONS_00026907 was generated through arrest at the G1‐ to S‐phase transition. A consistent result was found in SiHa cells. In addition, we found that the apoptosis capacity of HeLa cells transfected with shTCONS_00026907 was significantly increased when compared with the control group. A consistent result was found in SiHa cells (Fig. 2E). The migration and invasion capacity of HeLa cells transfected with shTCONS_00026907 was significantly decreased when compared with the control group. A consistent result was found in SiHa cells (Fig. 2F and G).

### TCONS_00026907 serving as a ceRNA accelerates ELK1 expression through inhibition of miR‐143‐5p and regulates C‐fos, Cyclin D1, and Bcl‐2 expression

**Figure 3:**
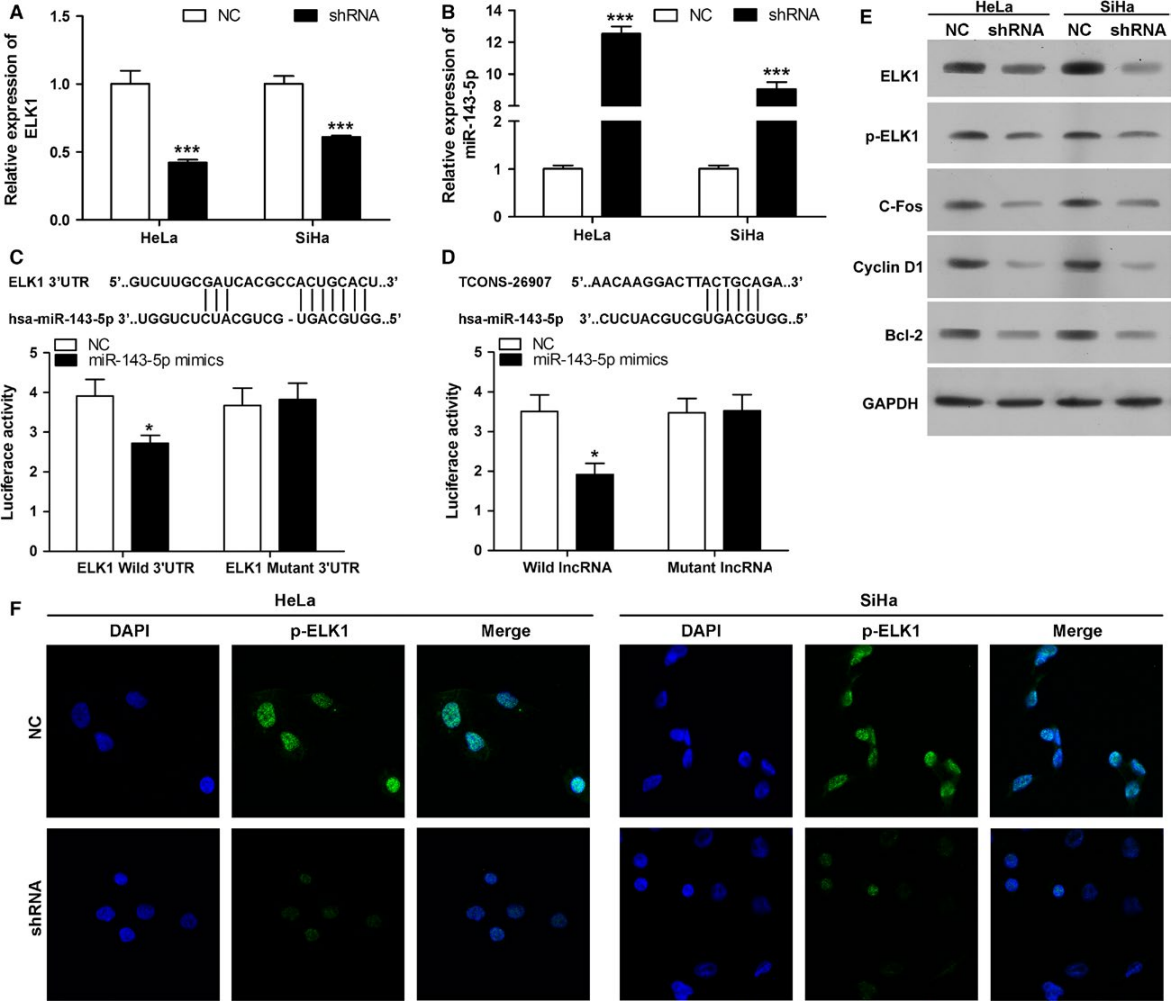
TCONS_00026907 promotes ELK1 expression through inhibition of miR‐143‐5p. (A, B) Quantitative real‐time PCR detected the mRNA expression level of ELK1 and miR‐143‐5p. Level of ELK1 (A) was increased, while miR‐143‐5p was inhibited in HeLa and SiHa cells transfected with shRNA (shRNA: targeting TCONS_00026907; ***P < 0.001). (C) The wild‐type ELK1 3′‐UTR or mutant ELK1 3′‐UTR‐containing vector was cotransfected into HeLa cells with miR‐143‐5p mimics. Mutation of ELK1 3′‐UTR suppressed the luciferase activity (*P < 0.05). (D) The wild‐type TCONS_00026907 sequence or mutant TCONS_00026907 sequence‐containing vector was cotransfected into HeLa cells with miR‐143‐5p mimics. Mutation of TCONS_00026907 sequence suppressed the luciferase activity (*P < 0.05). (E) Western blot analysis of ELK1, p‐ELK1, C‐fos, Cyclin D1 and Bcl‐2 proteins in HeLa and SiHa cells transfected with NC or shRNA. Protein level of ELK1, p‐ELK1, C‐fos, Cyclin D1 and Bcl‐2 were inhibited by shTCONS_00026907. (F) The expression level of p‐ELK1 was detected by the immunofluoresce assay. Merge: superimposed images of p‐ELK1 in green and nuclei (DAPI) in blue, 400× magnification.

To investigate the interactive relationship among TCONS_00026907, ELK1, and miR‐143‐5p, qRT‐PCR and luciferase report gene assay were performed. Our studies revealed that the mRNA expression level of ELK1 was significantly downregulated in HeLa and SiHa cells transfected with shTCONS_00026907 when compared with the control group (*P* < 0.001) (Fig. 3A). The results also indicated that the mRNA expression level of miR‐143‐5p was significantly upregulated in HeLa and SiHa cells transfected with shTCONS_00026907 when compared with the control group (*P* < 0.001) (Fig. 3B). The ELK1 sequence for the predicted binding of the miR‐143‐5p was cloned into the downstream of the luciferase gene. As shown in Figure 3C and D, decreases in the luciferase activity of the reporters in the wild‐type ELK1 3′‐UTR‐containing vectors and a partial sequence of TCONS_00026907‐containing vectors were observed in the presence of miR‐143‐5p. As shown in Figure 3E, the protein expression levels of ELK1, p‐ELK1, C‐fos, Cyclin D1, and Bcl‐2 were significantly downregulated in HeLa and SiHa cells transfected with shTCONS_00026907 when compared with the control group. Immunofluorescence assay results showed that the protein expression level of p‐ELK1 was decreased in HeLa and SiHa cells transfected with shTCONS_00026907 when compared with the control group (Fig. 3F).

### miR‐143‐5p inhibits the progression of cervical cells

**Figure 4:**
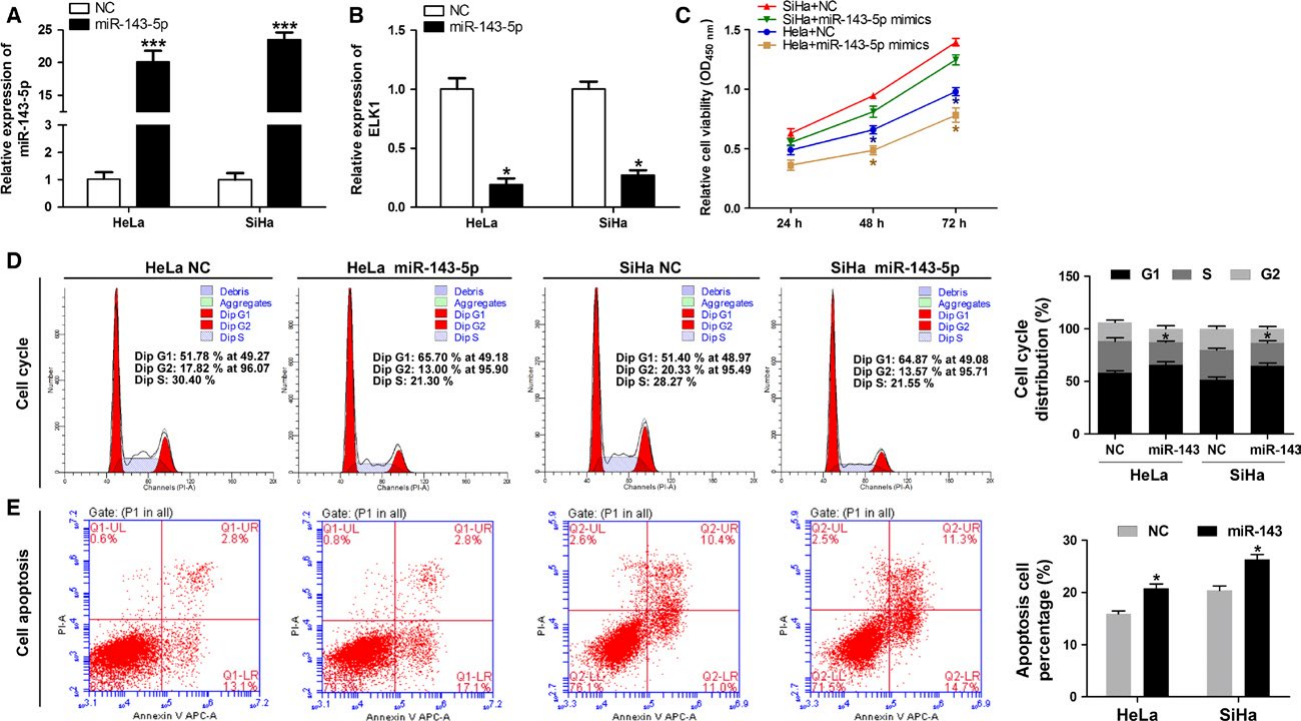
Overexpression of miR‐143‐5p inhibits the progression of cervical cells. HeLa and SiHa cells were transfected with negative control (NC) and miR‐143‐5p mimics. (A and B) The expression level of miR‐143‐5p and ELK1 were detected by Quantitative real‐time PCR. Level of miR‐143‐5p (A) was increased, while ELK1 was inhibited (*P < 0.05, ***P < 0.001). (C) Result of CCK‐8 assay revealed that proliferation of HeLa and SiHa cells is inhibited by overexpression of miR‐143‐5p (*P < 0.05). (D) Overexpression of miR‐143‐5p induced cell cycle arrest at G1/S phase (*P < 0.01). The cell cycle distribution was detected by flow cytometry. (E) Cell apoptosis was measured by Annexin V‐FITC/PI staining and flow cytometry and found that it was increased in HeLa and SiHa cells transfected with miR‐143‐5p mimics (*P < 0.01).

Firstly, qRT‐PCR was used to detect the mRNA expression levels of miR‐143‐5p and ELK1 in HeLa and SiHa cells transfected with negative control (NC) and miR‐143‐5p mimics. Our results indicated that the mRNA expression level of miR‐143‐5p was higher in HeLa cells and SiHa cells transfected with miR‐143‐5p mimics when compared with the NC (*P* < 0.001) (Fig. 4A). The mRNA expression level of ELK1 was lower in HeLa cells and SiHa cells transfected with miR‐143‐5p mimics when compared with the NC (*P* < 0.001) (Fig. 4B). Then we found that the proliferation ability was decreased in HeLa and SiHa cells transfected with miR‐143‐5p mimics when compared with the NC (Fig. 4C). Flow cytometry results revealed that the growth‐inhibiting effect of miR‐143‐5p mimics was generated during arrest at the G1‐phase to S‐phase transition (Fig. 4D). In addition, apoptosis capacity of HeLa and SiHa cells transfected with miR‐143‐5p mimics was significantly increased when compared with the control group (Fig. 4E).

### miR‐143‐5p inhibits the migration and invasion abilities of cervical cancer cells and regulates ELK1, p‐ELK1, C‐fos, Cyclin D1, and Bcl‐2 expression in vitro

**Figure 5:**
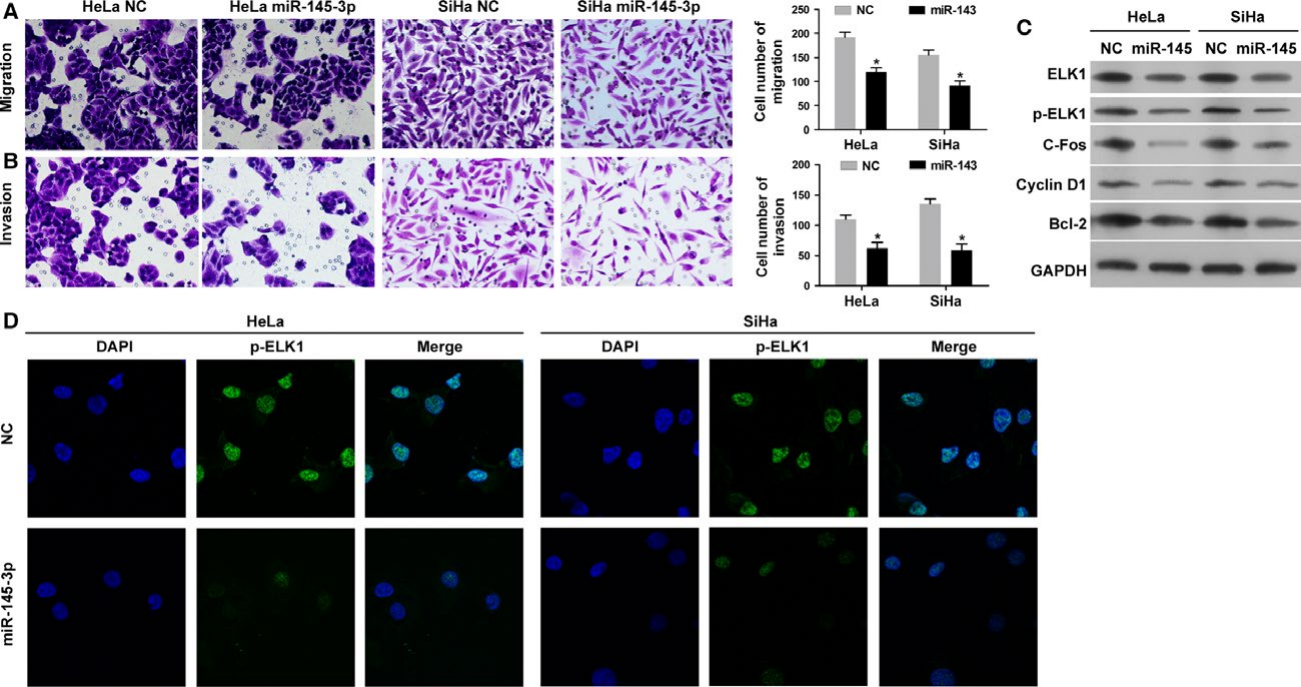
Overexpression of miR‐143‐5p inhibits the migration and invasion abilities of cervical cancer cells and regulates ELK1, p‐ELK1, C‐fos, Cyclin D1, and Bcl‐2 expression in vitro. (A and B) The abilities of migration and invasion were inhibited in HeLa and SiHa cells transfected miR‐143‐5p mimics (200× magnification), measured by Transwell assay(*P < 0.01). (C) Western blot analysis of ELK1, p‐ELK1, C‐fos, Cyclin D1 and Bcl‐2 proteins. Protein level of ELK1, p‐ELK1, C‐fos, Cyclin D1 and Bcl‐2 were inhibited by overexpression of miR‐143‐5p. (D) The protein level of p‐ELK1 was detected by the immunofluorescence assay. Merge: superimposed images of p‐ELK1 in green and nuclei (DAPI) in blue, 400× magnification.

The migration and invasion abilities of HeLa and SiHa cells transfected with miR‐143‐5p mimics and NC were measured by the Transwell assay. The results indicated that the migration and invasion capacities of HeLa and SiHa cells transfected with miR‐143‐5p mimics were significantly decreased when compared with the NC (Fig. 5A and B). As shown in Figure 5C, Western blot assay results indicated that the protein expression levels of ELK1, p‐ELK1, C‐fos, Cyclin D1, and Bcl‐2 were significantly downregulated in HeLa and SiHa cells transfected with miR‐143‐5p mimics when compared with the NC group. Immunofluorescence results showed that the protein expression level of p‐ELK1 was decreased in HeLa and SiHa cells transfected with miR‐143‐5p mimics when compared with the control group (Fig. 5D).

### Silencing of ELK1 by siRNA inhibits the progression of cervical cells

**Figure 6:**
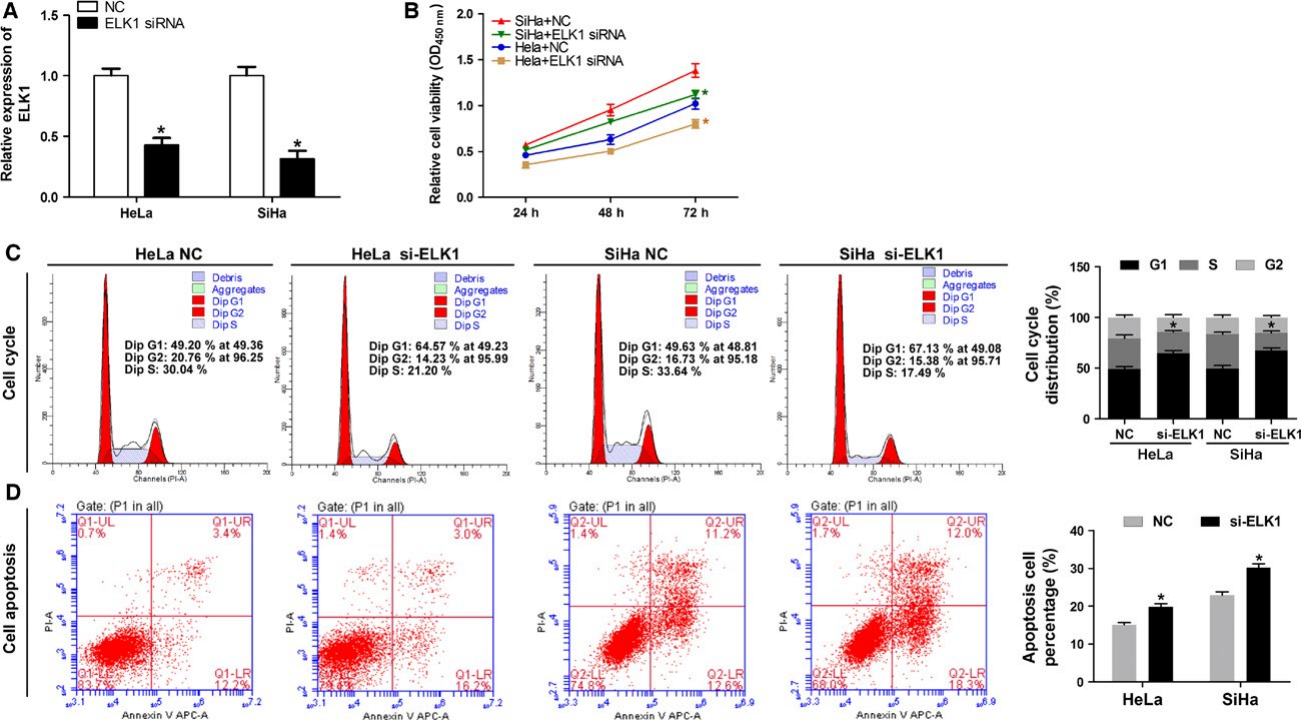
Silencing of ELK1 inhibits the progression of cervical cells. HeLa and SiHa cells were transfected with the negative control (NC) and ELK1 siRNA. (A) Quantitative real‐time PCR was used to detect the expression level. Level of ELK1 was down‐regulated by si‐ELK1 (*P < 0.05). (B) Result of CCK‐8 assay revealed that proliferation of HeLa and SiHa cells is inhibited by si‐ELK1(*P < 0.05). (C) Silencing of ELK1 induced cell cycle arrest at G1/S phase (*P < 0.01). The cell cycle distribution was detected by flow cytometry. (D) Cell apoptosis was increased in HeLa and SiHa cells transfected with ELK1 siRNA (*P < 0.01), measured by Annexin V ‐FITC/PI staining and flow cytometry.

QRT‐PCR results indicated that the mRNA expression level of ELK1 was lower in HeLa cells and SiHa cells transfected with ELK1 siRNA when compared with the NC (*P* < 0.05) (Fig. 6A). The CCK‐8 assay results indicated that the proliferation ability was decreased in HeLa and SiHa cells when they were transfected with ELK1 siRNA compared with the NC (Fig. 6B). Flow cytometry results indicated that the growth‐inhibiting effect of ELK1 siRNA is generated through the arrest at the G1‐phase to S‐phase transition (Fig. 6C). In addition, we found that the apoptosis capacity of HeLa and SiHa cells transfected with ELK1 siRNA was significantly increased when compared with the control group (Fig. 6D).

### Silencing of ELK1 by siRNA inhibits the migration and invasion abilities of cervical cancer cells and regulates ELK1, p‐ELK1, C‐fos, Cyclin D1, and Bcl‐2 expression in vitro

**Figure 7:**
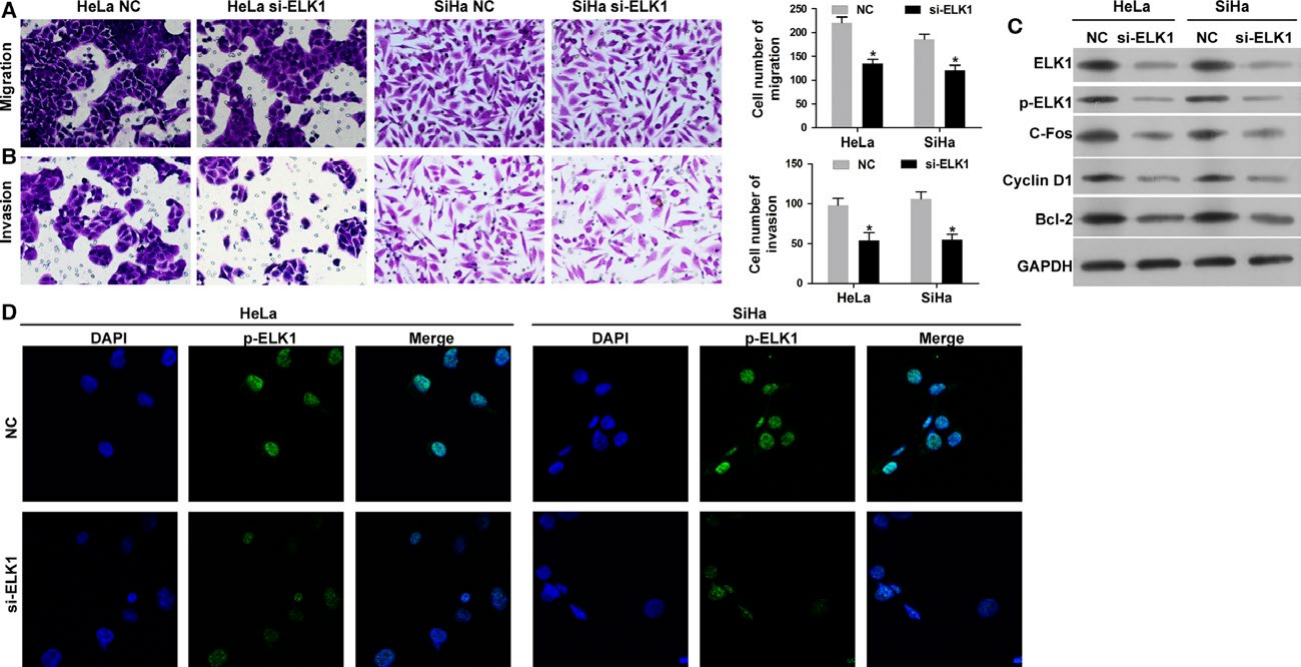
Silencing of ELK1 inhibits the migration and invasion abilities of cervical cancer cells and regulates ELK1, p‐ELK1, C‐fos, Cyclin D1, and Bcl‐2 expression in vitro. (A and B) The abilities of migration and invasion were inhibited in HeLa and SiHa cells transfected with ELK1 siRNA (*P < 0.01), measured by Transwell assay, 200× magnification. (C) Western blot analysis of ELK1, p‐ELK1, C‐fos, Cyclin D1, and Bcl‐2 proteins in HeLa and SiHa cells transfected with the NC and ELK1 siRNA. Protein level of ELK1, p‐ELK1, C‐fos, Cyclin D1 and Bcl‐2 were inhibited by si‐ELK1. (D) The protein expression level of p‐ELK1 was detected by the immunofluorescence assay in HeLa and SiHa cells transfected with the NC and ELK1 siRNA. Merge: superimposed images of p‐ELK1 in green and nuclei (DAPI) in blue, 400× magnification.

The results indicated that the migration and invasion capacities of HeLa and SiHa cells transfected with ELK1 siRNA were significantly decreased when compared with the NC (Fig. 7A and B). As showed in Figure 7C, Western blot assay results indicated that the protein expression levels of ELK1, p‐ELK1, C‐fos, Cyclin D1, and Bcl‐2 were significantly downregulated in HeLa and SiHa cells transfected with ELK1 siRNA when compared with the NC group. In addition, the immunofluorescence results indicated that the protein expression level of p‐ELK1 was decreased in HeLa and SiHa cells transfected with ELK1 siRNA when compared with the control group (Fig. 7D).

### Silencing of TCONS_00026907 expression suppresses the growth of cervical tumor and regulates ELK1, p‐ELK1, C‐fos, Cyclin D1, and Bcl‐2 expression in vivo

**Figure 8:**
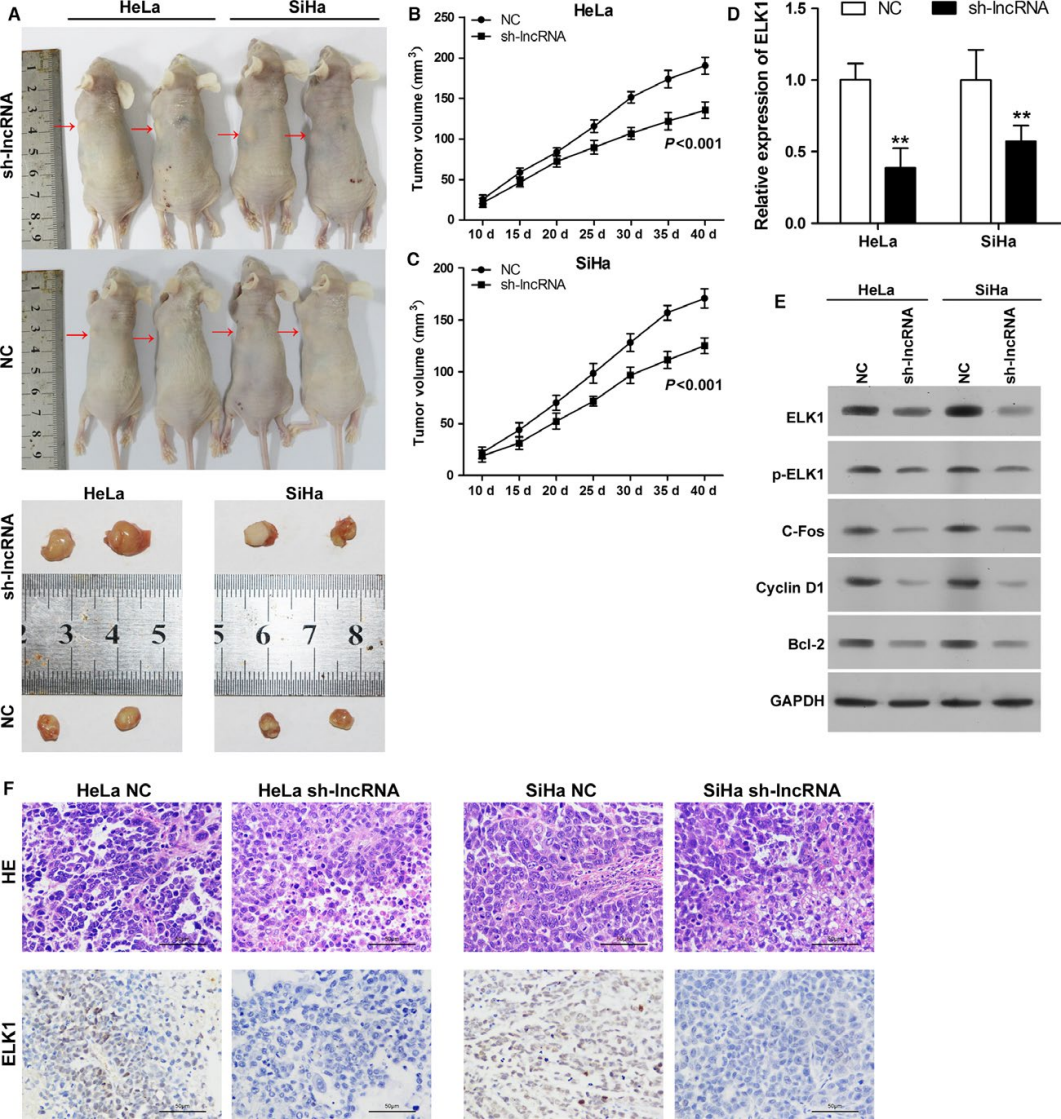
Silencing of TCONS_00026907 suppresses the growth of cervical tumors and regulates ELK1, p‐ELK1, C‐fos, Cyclin D1, and Bcl‐2 expression in vivo. Athymic mice were injected with HeLa and SiHa cells transfected with shTCONS_00026907. (A) Silencing TCONS_00026907 expression reduced the size of tumors (red arrow) in nude mice injected with HeLa and SiHa cells. (B) The tumor volume in nude mice injected with HeLa cells transfected with shRNA was reduced when compared with the negative control (NC) (*P* < 0.001). (C) The tumor volume in nude mice injected with SiHa cells transfected with shRNA was reduced when compared with the NC (*P* < 0.001). (D) The mRNA expression level of ELK1 was validated by quantitative real‐time PCR in tumors of HeLa and SiHa cells transfected with the NC or shRNA (**P < 0.01). (E) The protein expression levels of ELK1, p‐ELK1, C‐fos, Cyclin D1, and Bcl‐2 in tumors of HeLa and SiHa cells transfected with the NC or shRNA. GAPDH was used as a reference protein. (F) Representative images of H&E staining and immunohistochemistry detecting ELK1 in tumors of HeLa and SiHa cells transfected with the NC or shRNA. Levels of ELK1 in shRNA groups were down‐regulated.

To explore the effect of TCONS_00026907 on tumorigenesis in vivo, HeLa and SiHa cells transfected with shTCONS_00026907 were implanted subcutaneously into nude mice. The tumor was removed after 30 days (Fig. 8A). The tumor volume was calculated at 10, 15, 20, 25, 30, 35, and 40 days. The tumor volume was smaller in the mice injected with HeLa cells transfected with shTCONS_00026907 than in the mice injected with the NC (Fig. 8B). A consistent result was found in mice injected with SiHa cells (Fig. 8C). We found that the mRNA expression level of ELK1 was dramatically decreased in mice injected with HeLa and SiHa cells transfected with shTCONS_00026907 when compared with mice injected with the NC (Fig. 8D). We also found that the protein expression levels of ELK1, p‐ELK1, C‐fos, Cyclin D1, and Bcl‐2 were significantly downregulated in mice injected HeLa and SiHa cells transfected with shTCONS_00026907 when compared with mice injected with NC (Fig. 8E). H&E staining showed no significantly changes, however, immunohistochemistry results showed also confirmed that the expression of ELK1 was significantly suppressed in mice injected HeLa and SiHa cells transfected with shTCONS_00026907 when compared with mice injected with NC (Fig. 8F).

## Discussion

LncRNAs can regulate protein‐coding genes, transcriptional levels, post‐transcriptional levels, and can affect physiological processes 26, 27. Many earlier studies have shown that massive lncRNAs were associated with tumorigenesis, metastasis, prognosis, and diagnosis, and acted as oncogenes or cancer suppressor genes 26, 28. Therefore, the biological and molecular mechanisms of lncRNAs require more study to elucidate their role in cancer. In our study, we showed that TCONS_00026907 expression was higher in cervical cancer tissues than in adjacent noncancerous tissues. The survival rate was lower in the high expression group of TCONS_00026907 than in the low expression group. We also found that TCONS_00026907 accelerated the progression of the cervical cell cycle, proliferation, migration, and invasion, and inhibited cervical cancer cell apoptosis. Therefore, we speculated that TCONS_00026907 has an important effect in the development and progression of cervical cancer.

Cyclin D1 is a major regulator of the cell cycle progression in the G1 phase 29. Studies have shown that Bcl‐2 prevented apoptosis in an antioxidant pathway 30. We also demonstrated that TCONS_00026907 upregulated the protein expression level of Cyclin D1 and Bcl‐2 in vivo and in vitro. This further confirmed that TCONS_00026907 accelerated the progression of the cervical cell cycle and inhibited cervical cancer cell apoptosis.

A number of studies have shown that miRNAs play crucial roles in the biological process, including cell proliferation, metastasis, and inflammation by targeting mRNAs in numerous cancers 17, 31, 32. There are reports that various miRNAs such as miR‐21, miR‐214, miR‐218, and miR‐124 were related to the developmental process of cervical cancer 33, 34, 35, 36. The mechanisms and functions of miR‐143‐5p have been studied in gastric cancer 22 and prostate cancer 37. There are also studies indicating that the down regulation of miR‐143 is related to cervical squamous cancer 38. However, the molecular mechanism of miR‐143‐5p in cervical cancer is still not fully known. In our study, we showed that miR‐143‐5p down‐regulated the protein expression level of Cyclin D1 and Bcl‐2 in vivo and in vitro. Furthermore, we confirmed that miR‐143‐5p inhibited the progression of the cervical cell cycle, proliferation, migration, and invasion, and inhibited cervical cancer cell apoptosis through downregulation of the protein expression levels of ELK1, p‐ELK1, C‐fos, Cyclin D1, and Bcl‐2.

Recent studies have demonstrated that lncRNAs were closely related to the occurrence and development of various diseases 39, 40, 41. For example, the lncRNA H19, serving as a miRNA sponge, accelerates the development process of colorectal cancer 42. LncRNA‐MIAT, acting as a ceRNA, regulates microvascular dysfunction. LncRNA‐PTCSC3, serving as a target of miRNAs, affects the occurrence and development of thyroid cancer 43. In our study, we showed that when TCONS_00026907 was silenced, the mRNA expression level of miR‐143‐5p was significantly upregulated in HeLa and SiHa cells, indicating that TCONS_00026907 negatively regulated miR‐143‐5p transcription through targeted binding.

ELK1 acts as a member of the Ets family and the ternary complex factor (TCF) subfamily. Proteins of the TCF subfamily form a ternary complex by binding to the serum response factor and the serum response element in the promoter of the C‐fos 44. Our study indicated that ELK1 is a nuclear target for the ras‐raf‐MAPK signaling cascade 45. Our research showed that mutation of ELK1 3′‐UTR or mutation of TCONS_00026907 sequence to interrupt the binding of TCONS_00026907 and ELK1 3′‐UTR resulted in down‐regulation of luciferase reporter gene expression, suggesting that miR‐143‐5p negatively regulated ELK1 transcription through targeted binding. We also found that silencing ELK1 through siRNA inhibited the progression of the cervical cell cycle, proliferation, migration, and invasion, and inhibited cervical cancer cell apoptosis.

In short, this study indicated that TCONS_00026907 was upregulated in cervical cancer and TCONS_00026907 promoted the progression of cervical cancer through inhibition of miR‐143‐5p and promotion of ELK1.

## Conflict of Interest

The authors declare no conflict of interest.
